# Cancer research in the 57 Organisation of Islamic Cooperation (OIC) countries, 2008–17

**DOI:** 10.3332/ecancer.2020.1094

**Published:** 2020-08-28

**Authors:** Grant Lewison, Shoaib Fahad Hussain, Ping Guo, Richard Harding, Deborah Mukherji, Ghassan Abu Sittah, Ajay Aggarwal, Fouad Fouad, Nirmala Bhoo-Pathy, Omar Shamieh, Julie Torode, Tezer Kutluk, Richard Sullivan

**Affiliations:** 1 King's College London, Institute for Cancer Policy, Guy's Hospital, Great Maze Pond, London SE1 9RT, UK; 2 Conflict and Health Research Group, School of Security Studies, King’s College London, London SE1 9RT, UK; 3 School of Nursing, College of Medical and Dental Sciences, University of Birmingham, Edgbaston, Birmingham B15 2TT, UK; 4 Florence Nightingale Faculty of Nursing, Midwifery and Palliative Care, Cicely Saunders Institute, King's College London, London SE 9PJ, UK; 5 American University of Beirut Medical Center, Beirut, Lebanon; 6 American University of Beirut, Global Health Institute, Beirut, Lebanon; 7 King's College London, Institute for Cancer Policy, Guy's Hospital, Great Maze Pond, London SE1 9RT, UK; 8 American University of Beirut, Faculty of Health Science, Beirut, Lebanon; 9 Centre for Epidemiology and Evidence-Based Practice, Faculty of Medicine, University of Malaya, 50603, Lembah Pantai, Kuala Lumpur, Malaysia; 10 King Hussein Cancer Centre, Amman, Jordan; 11 School of Medicine, the University of Jordan, Amman, Jordan; 12 Union for International Cancer Control (UICC), Avenue Giuseppe Motta 31-33, 1202, Geneva, Switzerland; 13 Haceteppe University, Ankara 06100, Turkey; 14 King's College London, Institute for Cancer Policy, Guy's Hospital, Great Maze Pond, London SE1 9RT, UK

**Keywords:** cancer research, Organisation of Islamic Cooperation, cancer anatomical sites, research types, funding, citations

## Abstract

**Background and objectives:**

The 57 countries of the Organisation of Islamic Cooperation (OIC) are experiencing rapid increases in their burden of cancer. The First Ladies Against Cancer meeting at the 2016 OIC meeting in Istanbul committed to the importance of cancer control and the need for more evidence to support national cancer control planning (NCCP). Strong research systems are a crucial aspect of NCCP, but few data exist to support policy-makers across this political grouping

**Methodology:**

We identified all cancer research papers from OIC countries in the Web of Science from 2008 to 2017 with a filter based on journal names and title words, with high precision and recall. We analysed the country outputs, the cancer sites investigated, the types of research, sources of funding and the citations to the papers.

**Results:**

There were 49,712 cancer research papers over this period. The leading countries in terms of output were Turkey, Iran, Egypt and Malaysia, but the most cited papers were from Qatar, Indonesia and Saudi Arabia. International collaboration was low, except in Qatar and the United Arab Emirates. The site-specific cancers accounting for most research were breast and blood, correlating with their disease burden in the OIC countries, but lung, cervical and oesophageal cancers were relatively under-researched. Most funding from within the OIC countries was from their own university sector.

**Conclusion:**

Cancer is seriously under-researched in most of the OIC countries. This will undermine the ability of these countries and OIC as a whole to deliver on better cancer control for their populations. New policies, OIC leadership and funding are urgently needed to address this situation.

## Introduction

The Organisation of Islamic Cooperation (OIC) is the second largest intergovernmental organisation after the United Nations (UN) with a membership of 57 countries and 5 observer states across four continents, but primarily in Asia and Africa (27 in each), as shown in Table 1. The OIC aims to represent the collective political and socio-economic interests of the Muslim world [1, 2]. The organisation also includes efforts to enhance cooperation between member states in healthcare, technology, research and development and education although it does not have a specific focus on cancer control [3]. OIC member states collectively constitute over 1.8 billion people, i.e., approximately 23% of the world’s population. With a population growth rate of 1.86% between 2010 and 2015, this proportion is projected to increase with rapid ageing that makes cancer control in the next decade a crucial health domain [4].

The OIC has a diverse and contrasting socio-economic and human development index (HDI) profile, encompassing some of the least developed regions in the world, e.g., Sub-Saharan Africa and some of the wealthiest and most developed countries in the Arabian Gulf. It also faces challenges from conflicts, e.g., Syria and Yemen. OIC member states collectively host the world’s largest demographically transitioned refugee populations, for which cancer control is a significant issue [5]. In light of these challenges, the OIC and subsidiary bodies such as the Islamic Development Bank (IsDB) have galvanised support among Muslim majority countries for global health objectives [16, 7]. Initiatives such as the Global Muslim Philanthropy Fund for Children [8] and the OIC 2025: Programme of Action have also been launched to enhance the efforts by OIC member states to achieve the sustainable development goals including cancer control [9].

The importance of cancer control for OIC becomes apparent because of 18.1 million new cancer global diagnoses in 2018, and 10% were in OIC member states [10, 11], as were 12% of global cancer deaths [4]. The rising burden of cancer was acknowledged at the 13th OIC Summit in Istanbul in April 2016 with the Istanbul Declaration against cancer under the auspices of the First Ladies’ Leadership on Cancer Control [4]. As a part of the Istanbul Declaration, the Research for Health in Conflict partnership (r4hc-mena.org) committed to providing research evidence to inform not only the widely distinct political research economies of OIC member states but also key research funders such as the IsDB and the African Development Bank [12]. The main objective of this analysis is to provide information about one of the critical pillars of cancer control, namely research. Insights into relative strengths and weaknesses of cancer research across the OIC can be used by both national policy-makers and more widely within supranational OIC strategies to support new approaches to building research ecosystems.

## Methodology

### Search strategy

Articles and reviews in cancer research in 2008–17 with an address in one of the 57 OIC countries were identified in the Web of Science (WoS, © Clarivate Analytics). They were identified by means of a previously validated search filter (ONCOL) based on 185 specialist cancer journals and 323 title words or phrases, which included the names of cancers, drugs used to treat them and genes that increased (or decreased) the risk of cancer [13, 14]. Papers were selected if they were in a cancer journal or had one or more of the listed words or phrases or both. [Cancer journals were included if 90% (or more) of their papers had one of the listed title words.] The precision (specificity) of the filter was *p* = 0.95 and the recall (sensitivity) was *r* = 0.98, so the true number of cancer papers was 0.95/0.98 = 0.97 times the apparent number. All the papers identified by the filter were retained, and their details were downloaded to a spreadsheet and saved for 50 that had been retracted. There was no language restriction.

This was carried out in September 2018, by which time the tally of 2017 papers would have been effectively complete. The 5-year citation scores (actual citation impact, ACI) for the papers from 6 years, 2008–13, were also determined from the WoS separately and copied and pasted to the file of papers.

### Analysis of countries and subjects

The addresses on the papers were analysed by means of a Visual Basic for Applications program to show the fractional counts of the countries. For example, a paper with two Turkish addresses and one from France would be classed as TR = 0.67 and FR = 0.33. To put the research outputs of the different countries in context, they were plotted against the healthcare expenditures of the leading OIC states. [We also tried plotting them against the wealth and populations of the OIC countries.] As the outputs of the leading OIC states had been increasing quite rapidly in recent years and as, in 2015, the WoS was expanded to process many additional journals published outside North America and Western Europe, we used outputs in 2015–17 and compared them with healthcare expenditures for 2015.

We also applied two special filter programs to the papers’ titles and journal names in order to classify them by the anatomical cancer site(s) investigated by the authors and the type of research (e.g., genetics, surgery and palliative care) [14]. We used a similar methodology to identify papers that concerned the clinical trials or paediatrics. We compared the overall OIC outputs on major cancer sites with the relative collective disease burden in DALYs from cancers on each site in 2010. [Data for the disease burden in 2015 are available, but the amount of research in 2008–17 would not have been much influenced by them.] Outputs on different anatomical sites or research types were also cross-tabulated by the fractional counts of the leading OIC countries (i.e., the 15 with the largest outputs of cancer research papers) so as to show which ones were relatively specialising on particular sites or research types.

We also wished to see the amount of international collaboration in cancer research by the OIC countries. For this purpose, we calculated the fractional count contributions of the leading OIC countries to their papers, and the corresponding contributions by other OIC countries, by Canada and the USA, the EUR31 countries (the 27 member states of the European Union + Iceland, Norway, Switzerland and the UK) and the Rest of the World.

### Analysis of funding

A further analysis was of the funding of these cancer research papers. Since 2009, the Science Citation Index, a major component of the Web of Science, has recorded the funding acknowledgments on the papers it includes. However, the names of the individual explicit funders are given in a number of different formats, so we use three-character (trigraph) codes to identify them, together with two digraph codes to connote their sector (government, non-profit, commercial and international) and their nationality [15]. We recorded the numbers of funders per paper and the percentage of papers, which acknowledged explicit funding, for the OIC papers with and without third-country collaboration as a function of time. We also coded for the analysis of the major funding sources in 10 non-OIC countries who collaborated on these papers and also the main governmental and academic (university) funding sources in 10 leading OIC countries.

### Analysis of citations

The citation counts of the papers in the first 5 years beginning with the year of their publication, ACI, were multiplied by the fractional contributions of each country to each paper, and the totals were then divided by the fractional counts of the countries’ contributions. The papers were also sorted by their ACI values, and the top 5% of the whole cohort for the 6 years from 2008 to 2013 identified; they were cited 29 times or more. The percentages of each country’s citable papers that received this number of citations or more, on a fractional count basis, were then calculated as percentages, divided by the average for the OIC of 5% and then multiplied by 100 to give a ‘world scale’ value [16]. This gives an alternative ranking of the OIC countries based on their presence among the most cited papers although this is normally fairly similar to one based on mean ACI values.

## Results

### Cancer research outputs

**Figure 1.**
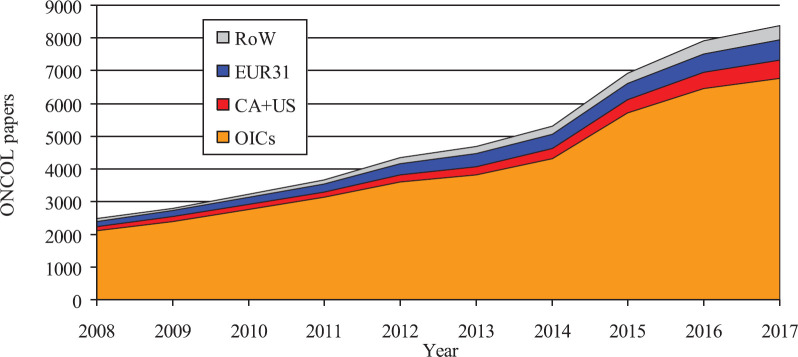
Outputs of cancer research papers in the WoS from the OIC countries, 2008–17, showing the contributions from Canada + USA, the EUR31 countries and the rest of the World (RoW). Note the big increase from 2014 to 2015 caused by the increase in journal coverage of the WoS.

The total number of papers in the file was 49,712 over the decade 2008–17, of which authors from OIC countries contributed a total of 41,064 (83%), and the remainder came from EUR31 countries (7.4%), Canada and the USA (5.7%) and the rest of the World (4.3%). The gross output of OIC cancer research papers rose from 2470 in 2008 to 8,387 in 2017 or by a factor of 3.4. However, the biggest year-on-year increase was from 5,297 in 2014 to 6,923 in 2015 when the WoS increased its coverage of journals from OIC countries (and others), as shown in Figure 1.

Overall, the top four cancer research-active OIC countries were Turkey (TR), with 15,269 papers in 2008–17, Iran (IR with 8,374), Egypt (EG with 4,330) and Malaysia (MY with 2,466). The output from most countries grew at about 14% *per annum* over the decade, but it was much higher in Qatar (QA +35% p.a.) and Indonesia (ID +29% p.a.) and lower in Turkey (+8.2% p.a.), Nigeria (NG +7.5% p.a.) and, especially, Tunisia (TN +2.3% p.a.) and actually *negative* in Kuwait (KW, –3.8% p.a. [For most OIC countries with small outputs, an annual percentage increase is not meaningful.]

**Figure 2.**
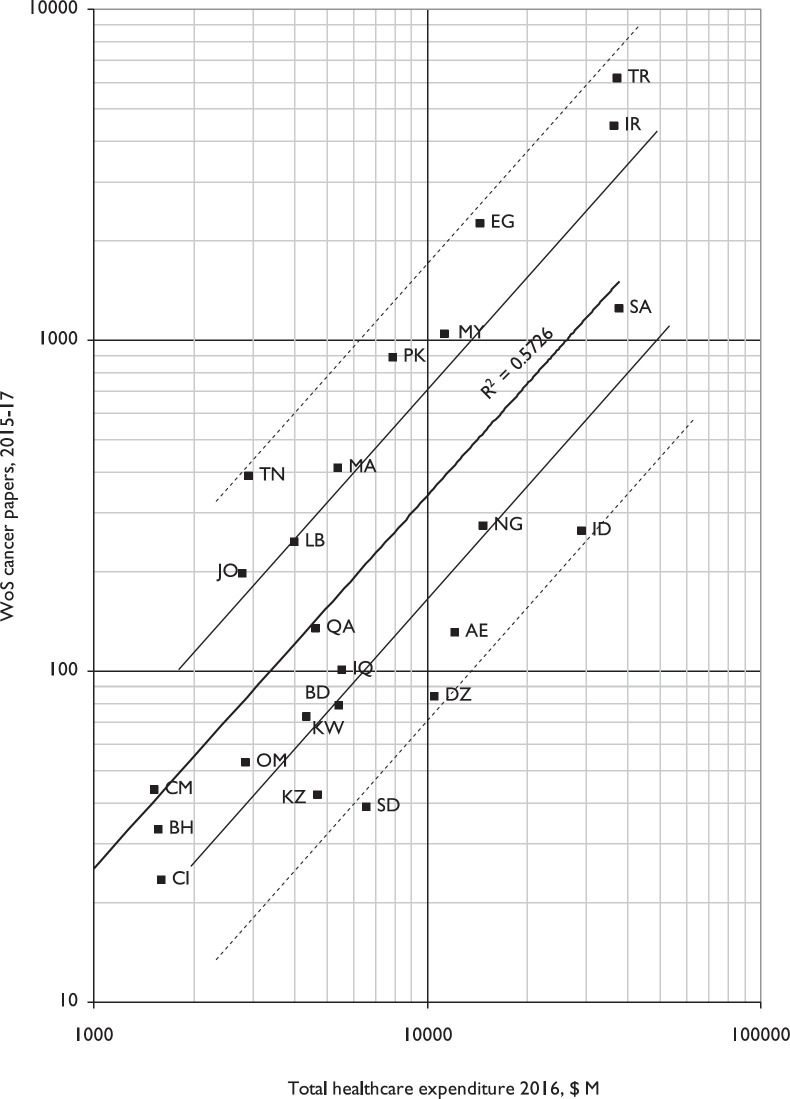
Plot of cancer research papers from 24 leading OIC countries in 2015-17, fractional counts, against their healthcare expenditures in 2016, US $ million. Log-log scales. For ISO2 codes, see Table 1.

A comparison of country outputs in 2015–17, for those with at least 10 papers, with their healthcare expenditures in 2015, is shown in Figure 2 (Libya and Syria are omitted as GDP data were not available). Tunisia, Egypt and Turkey were relatively the most productive, with more than four times the output that would be expected based on the least-squares correlation line, whereas Sudan, Algeria and Indonesia were relatively the least research-active, compared with their national healthcare expenditures. The correlation with a power-law least squares line (based on all the OIC countries, for which the data were available) is positive and fairly good (*r*^2^ = 0.57) It is much better than for a plot of research outputs against GDP (for which *r*^2^ = 0.29), against population (*r*^2^ = 0.03), or with a plot of papers per million population against the countries’ HDI for which *r*^2^ = 0.06.

### International collaboration

**Figure 3.**
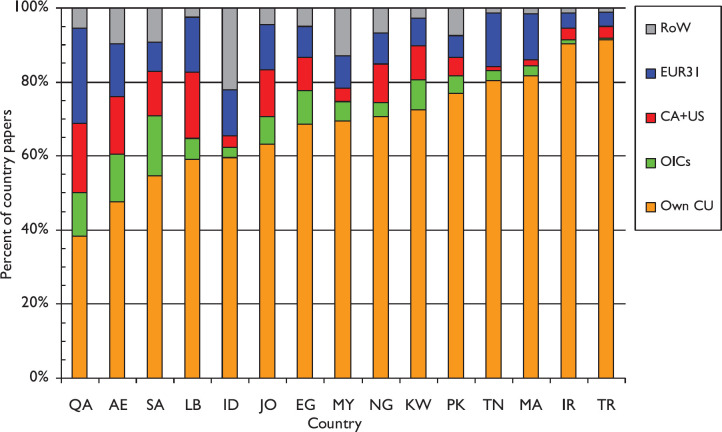
The proportion of outputs through collaboration by the leading OIC countries with others in the group, Canada and the USA, the EUR31 countries and the rest of the World (RoW), for 2008–17. For ISO2 country codes, see Table 1.

Figure 3 shows the amounts of international collaboration for the 15 leading OIC countries, ordered by the percentage of foreign contributions to their papers. These range from nearly 62% for Qatar (QA) down to 9% for Turkey. The OIC countries collaborate very little with each other. For these 15 countries, only 4.4% of their outputs come from others in the total group of the 56 other countries, compared with Canada and the USA, and the EUR31 countries. The largest intra-OIC collaborations are for Saudi Arabia (SA, 16.3%), the United Arab Emirates (AE, 12.8%), Qatar (QA, 11.7%) and Egypt (EG, 9.1%).

### Specialisation of OIC countries on cancer sites and research types

**Figure 4.**
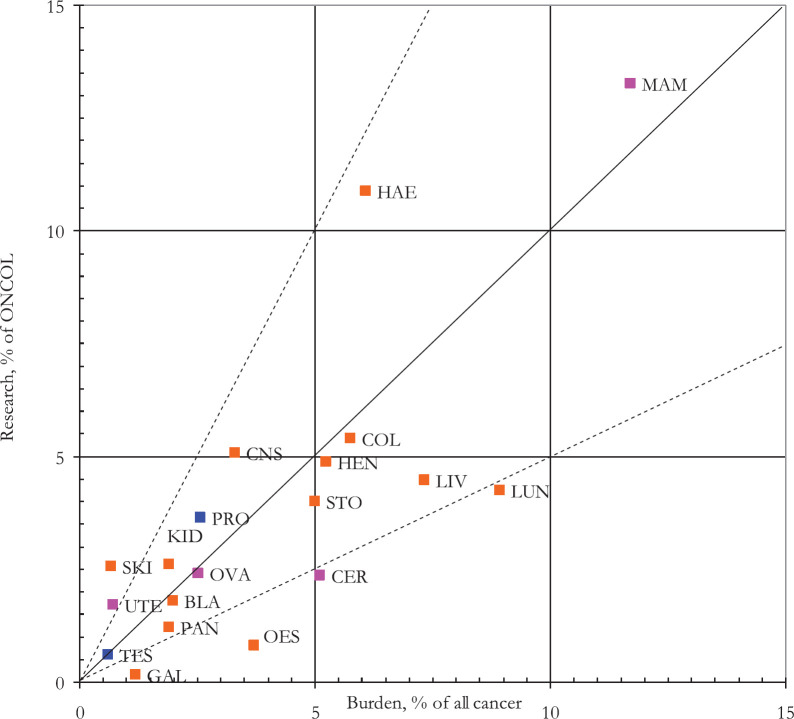
Percentages of all OIC cancer research papers, 2008–17, on individual cancer sites (integer counts) plotted against their collective burden (in DALYs; WHO data for 2010). (Blue spots: male cancers; pink spots: female cancers.) BLA=Bladder; CER=Cervix; CNS=Central nervous system; COL=Colon and rectum; GAL = gall bladder; HAE = Haematology; HEN=Head and Neck; KID = Kidney; LIV = Liver; LUN = Lung; MAM = Breast; OES = Oesophagus; OVA = Ovary; PAN = Pancreas; PRO = Prostate; SKI = Skin (incl. Melanoma); STO = Stomach; TES = Testicles; UTE = Uterus.

Figure 4 shows the number of papers (integer counts) on each of the major cancer sites, together with their disease burden for the OIC countries in 2010. The different OIC countries varied in the percentages of their overall cancer research outputs that were relevant to each cancer site, relative to the percentages shown in Figure 4. There were 2,985 papers on children’s cancer (6.0%), reflecting the relatively high burden from paediatric cancers in the OIC countries (8.4% of their overall cancer burden in DALYs, compared with 2.4% in European countries).

Table 2 shows the relative concentration of the leading 15 OIC countries on research on the different anatomical sites. The figures compare each country’s fractional production compared with that of all 57 OIC countries. For example, breast cancer (MAM) accounted for 13.3% of all OIC cancer papers and Malaysia (MY), which published a total of 2,466 cancer research papers, would have been expected to publish 327 on breast cancer. Its actual total was 501 papers, 1.53 times the expected value, and the difference was statistically highly significant (*p* < 0.01% on the Poisson distribution with one degree of freedom). Gynaecological cancers (GYN) were calculated as the total of cervical, fallopian tube, ovarian, uterine and vulva cancers. The cells are tinted in five bands, chosen to show minor (> √2 or < 1/√2) and major (> 2 or < 0.5) departures from the ‘norm’ value of unity.

**Figure 5.**
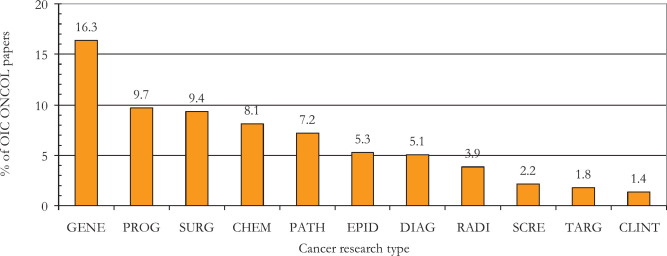
Percentages of OIC countries’ cancer research, 2008–17, in 12 domains. GENE = genetics; PROG = prognosis; CHEM = chemotherapy; SURG = surgery; PATH = pathology; EPID = epidemiology; DIAG = diagnosis; RADI = radiotherapy; SCRE = screening; CLINT = clinical trials.; PALL = palliative care; QUAL = quality of life.

Figure 5 shows that the type of research most published by the 57 OIC countries as a group was genetics, with over 16% of the total, followed by prognosis (biomarker-related research) and chemotherapy with 10% and surgery with 9%. Radiotherapy research accounted for just 4% of papers. Palliative care research only represented 1.3% of the total OIC output; it was concerned primarily with breast cancer patients (120 out of 657 papers, or 18%) and children (78 papers, 12%). The distribution of OIC cancer research papers between the different research types is almost exactly the same as that in Europe (EUR31; *r*^2^ = 0.97) [14]. Table 3 shows the relative commitment to particular domains of cancer research across the 15 research-active countries, which is prepared similarly to Table 2.

There were only 684 clinical trial papers (1.4%), for all four phases: this is fewer than the world average (3.2%) in the same years and much below the percentages in Canada (7.8%) and the four leading European countries (France, Germany, Italy and the UK = 9.6%). In the nine years (2009-17) for which funding data in the WoS were available, only 314 out of 654 clinical trial papers (48%) had a financial acknowledgment, and of these, only 111 acknowledged support from a pharmaceutical or biotech company (17% of the total).

### Analysis of funding acknowledgments

The percentage of OIC cancer research papers in 2009–17 without contributions from non-OIC countries rose from 16% in 2009 to 32% in 2017: this rise is partly an artefact because the WoS increased its coverage of acknowledgments over the period. The corresponding percentages for papers with third-country collaboration rose from 54% to 65%. It is also clear that the latter have many more funding acknowledgments, by a factor of two, or even more in the early years of the study period. The mean number of funders was only 1.3 for the non-collaborative papers but rose steadily from 2.6 to 4.4 for the ones with a non-OIC co-author.

It also appeared that there was relatively little explicit contestable funding for the OIC researchers, and their predominant sources of support were from their university funds rather than from government agencies running competitive support schemes (Table 4). This was particularly true for Iran, where universities were acknowledged more than ten times as often as government agencies. However, a few OIC countries received more support from government departments and agencies than they did from universities (notably Tunisia and Pakistan, but also Indonesia and Bangladesh). None of them had any significant sources of charitable funding (either collecting charities or endowed foundations) which are common in Western Europe, North America and Australasia. The African Development Bank was acknowledged on only ten papers and the IsDB on seven of them. The leading non-OIC funders were the US government departments and agencies (*e.g*., the National Cancer Institute), who, in total, supported about 7.6% of all the OIC papers and charitable sources (e.g., Cancer Research UK and the Wellcome Trust). A support from industrial companies (led by Hoffman La Roche s.a., Novartis s.a., Sanofi-Aventis s.a. and Merck Inc.) only amounted to about 1.9% of the papers, compared with 6.6% in Europe [14].

### Analysis of citations

The final analysis was of citations to papers published in 2008–13 for the OIC countries. The mean ACI for most of the individual OIC countries is below the average value (ACI = 9.0) for the whole set of OIC cancer research papers and well below the world average 5-year citation score of 16 cites in these years. The results are shown in Table 5, with the countries ranked by their ‘World-Scale’ performance, i.e., the proportion of their papers with enough cites to put them in the top 5% of all the OIC papers, which is 29 cites or more. This is calculated relative to the mean of 100. WS is below average for IR and all the countries below it in the table, but even the better performing countries’ presence among the top 5% is not significant on the Poisson distribution with one d/f. Indonesia (ID) performs well in terms of mean ACI probably because 27% of its citable papers are co-authored with the Netherlands and 29% with Japan. Qatar (QA) also does well, as 89% of its citable papers are internationally co-authored, two-fifths of them with the USA.

## Discussion

As a political group, the OIC countries shoulder a significant and growing global burden of new cancer cases and cancer deaths [11]. The ratio of deaths from cancer to the number of new cases is 0.62 in the OIC countries as a group but only 0.46 in Europe and 0.29 in North America and Oceania. These data show that treatment in the OIC countries, particularly those in Africa, lags significantly behind that available in other parts of the world. The burden of cancer is only set to grow as countries complete their demographic and epidemiological transitions. Furthermore, globalisation also increases OIC countries’ risk from increasing exposure to procancer risk factors such as tobacco [6, 7].

National cancer research activity is important for two reasons. First, it enables these countries to apply and adapt international treatment standards based on national burden and practice, and second, it can provide better career prospects to aid the retention of research-active cancer healthcare workers in the OIC and reduce the amount of emigration [18]. The provision of a high-quality medical research culture in a country will also attract international collaborations, which have become increasingly important for complex large-scale clinical trials. As a political group, the OIC is well placed in terms of both advocacies for cancer control across the vastly different member countries but also to leverage capacity and capability funding to build both mature and nascent cancer research systems within the National Cancer Control Plans. This will require radically different policy approaches.

The 57 OIC countries are very heterogeneous in terms of their cancer research outputs, with a factor of almost 90,000 between the most productive (Turkey, TR) and the least (Turkmenistan, TM). The developed countries of Western Europe show a close correlation between their cancer research outputs and their GDPs as do the countries of Eastern Europe and the former Soviet Union [14, 19]. However, the correlation for the OIC countries between GDP and cancer research output is much weaker, and the research correlates better with healthcare expenditure (Figure 2). Again, this speaks to the need for a more formal central co-ordination and advocacy role for the OIC.

The analysis suggests that there are two broad groups of countries in terms of cancer research. Some publish many papers, notably in TR, Iran (IR), Egypt (EG) and Malaysia (MY) and far fewer in the UAE (AE), Indonesia (ID) and Sudan (SD). However, some countries well below the regression line have been severely affected by conflict, notably Kuwait and Yemen. In addition, OIC countries in Sub-Saharan Africa [Nigeria (NG), Sudan (SD), Côte d›Ivoire (CI) and Burkina Faso (BF)] are also performing well below what would be expected. For the latter three which fall into the fragile and low-income categories, this is to be expected, but Nigeria stands out as a key example of where there is an urgent need to significantly improve cancer research for a major emerging power.

The strong showing of the OIC countries in genetics research (Figure 5), which is relatively basic, which is relatively basic, seems at odds with what is needed to inform national cancer control planning. Instead, it reflects the Western-dominated bias towards this research domain. One might have expected proportionately more research on screening and diagnosis and the means of treatment, such as surgery and radiotherapy. The widely different relative concentration of some countries on particular research types shown in Table 3 suggests that more international collaboration would be advantageous for those countries that are under-researching them.

We wondered if this distortion of the research agenda in favour of a pattern more suited to industrial countries might be caused by the research cadres in some of the small middle eastern countries having a high proportion of immigrants. We, therefore, listed the names of the cancer researchers in 2015–17 in five countries: the UAE, Jordan, Kuwait, Qatar and Oman. With the aid of a proprietary database of names, Origins, these were classed in 10 geographical groups. Table 6 shows the numbers of individuals in each group, after those listed with only initials were amalgamated with those whose given names were also given in the WoS. The allocation to the groups, “Levant including Turkey”, “Maghreb plus Egypt” and “Other Muslim countries”, was disputable as their Arab-sounding names appeared rather similar, and indeed, only about 8% of the names were classed as coming from the five countries. It is clear that immigrants only represented about a quarter of the total—western Europeans being the most numerous at 10% overall but 14% in the UAE and 17% in Qatar. However, Table 3 shows that genetics was not a priority for research in either country.

The funding analysis, though preliminary, showed that there was very little explicit contestable funding available in the OIC countries (except to their non-OIC partners). This is not a recipe for the production of high-impact research. It may also be one of the reasons for the lack of international collaboration shown in Figure 3, except for Qatar, the UAE and Saudi Arabia, whose generous funding has attracted foreign partners. It may also account for the relatively poor citation performance on the global scale. The overall citation performance of the OIC countries (ACI = 9.0 cites in five years) is significantly lower than the global average, which was 16.3 in 2009. The relatively low citation rate for Turkey and Iran, the two largest OIC countries in terms of output, warrants further examination by national policy-makers. Notably, international collaborations in these countries are scarce, accounting for less than 10% of their output, but there is also a lack of contestable funding sources.

Many OIC countries have had to allocate resources to combat more prevalent communicable diseases and NCDs such as cardiovascular disease. They have younger populations and have failed to respond adequately to cancer, which is catching up as a major driver of mortality. Limited resources are made available at the national level to fund cancer research in these countries and, even when present, are channelled to genetic research. Clinical research in cancer is critical in these countries but severely lacking. Late-stage cancer diagnosis remains a major problem, and research into detection encompassing early diagnosis and screening should be prioritised. Patients diagnosed with cancer in these settings often get lost navigating the healthcare system, and local solutions may only be available if research is undertaken in this area [20, 21]. Cancer survival in many OIC countries also appears to be significantly lower than many equivalent non-OIC countries [22], and the importance of improving cancer research ecosystems must be the key future strategy. Furthermore, cancer survivorship care is neglected as most health systems tend to be focussed on the provision of treatment and the management of short-term side effects from adjuvant cancer therapy. Findings from the ASEAN Costs in Oncology (ACTION) study, which included Malaysia and Indonesia, have shown that patients surviving cancer in the region continued to have persistently impaired health-related quality of life and high levels of psychological distress [23, 24]. Again, these are domains that lack any research focus across most OIC countries.

## Limitations of this study

One of the main limitations in this study is that the data were obtained from a single source, which may be less comprehensive in its coverage of papers from these countries than some others, such as MedLine or SCOPUS. However, MedLine only records the address of the corresponding author so would have failed to show the lack of internationalism of the OIC countries and would have omitted those, where the corresponding author was in a non-OIC country. The choice of the WoS rather than SCOPUS was primarily because of the availability of extensive software that enabled us to carry out the analysis efficiently. Moreover, the presence of additional papers in possibly less influential journals would have adversely affected the OIC countries’ citation scores.

## Conclusion

The analysis demonstrates that cancer care remains under-researched across much of the OIC, and urgent action is needed to bolster cancer control. The Istanbul Declaration provided a useful framework to unite OIC member states, prioritise cancer in their health and development agenda and increase collaboration between member states and international organisations such as the UN and the World Health Organisation (WHO). Ongoing capacity-building efforts, e.g., Research for Health in Conflict (r4hc-mena.org) including cancer prevention programmes as well as technical and material assistance to improve early detection, diagnosis and treatment are welcome, but it is imperative that the significant deficits in cancer research output are addressed now. The OIC as a political body should strengthen existing collaborative networks, form new research and health policy partnerships and work collectively to address key research and policy issues both at national and trans-OIC levels.

## Conflicts of interest

The authors declare no conflicts of interest.

## Funding

This publication is funded through the UK Research and Innovation GCRF RESEARCH FOR HEALTH IN CONFLICT (R4HC-MENA); developing capability, partnerships and research in the Middle and Near East (MENA) ES/P010962/1.

## Figures and Tables

**Table 1.** List of the OIC countries, with their ISO2 codes, populations in 2015 (million) and their GDPs *per caput* in that year (US dollars).

Countries	ISO2	Pop	GDP/caput	Countries	ISO2	Pop	GDP/caput
U Arab Emirates	AE	8.1	44640	Morocco	MA	32.6	3080
Afghanistan	AF	33.4	670	Mali	ML	16.3	820
Albania	AL	3.2	4450	Mauritania	MR	3.6	1370
Azerbaijan	AZ	9.4	7600	Maldives	MV	0.4	8260
Bangladesh	BD	152.4	1080	Malaysia	MY	29.3	11120
Burkina Faso	BF	17.5	690	Mozambique	MZ	24.5	620
Bahrain	BH	1.4	20860	Niger	NE	16.6	420
Benin	BJ	9.4	890	Nigeria	NG	167	2970
Brunei	BN	0.4	31590	Oman	OM	2.9	18340
Cote d’Ivoire	CI	20.6	1450	Pakistan	PK	180	1400
Cameroon	CM	20.5	1350	Palestine	PS	4.6	3090
Djibouti	DJ	0.9	1030	Qatar	QA	1.9	92320
Algeria	DZ	36.5	5490	Saudi Arabia	SA	28.7	25500
Egypt	EG	84.0	3210	Sudan	SD	37.2	1710
Gabon	GA	1.6	10410	Sierra Leone	SL	6.1	770
Guinea Bissau	GW	1.6	590	Senegal	SN	13.1	1025
Gambia	GM	1.8	460	Somalia	SO	9.8	728
Guinea	GN	10.5	470	Surinam	SR	0.5	9590
Guyana	GY	0.8	4036	Syria	SY	21.1	1850
Indonesia	ID	245	3630	Chad	TD	11.8	980
Iraq	IQ	33.7	6410	Togo	TG	6.3	550
Iran	IR	75.6	6550	Tajikistan	TJ	7.1	1350
Jordan	JO	6.5	4590	Turkmenistan	TM	5.2	7530
Kyrgyzstan	KG	5.4	1260	Tunisia	TN	10.7	4035
Comoros	KM	1.0	555	Turkey	TR	74.5	10630
Kuwait	KW	2.9	49300	Uganda	UG	35.6	670
Kazakhstan	KZ	16.4	12475	Uzbekistan	UZ	28.1	2070
Lebanon	LB	4.3	8120	Yemen	YE	25.6	1330
Libya	LY	6.5	7820				

**Table 2.** Relative output within cancer research to work on 11 cancer sites (codes below Figure 2) by the leading 15 countries (see Table 1), 2008–17. Cells where this ratio > 2.0 tinted green; >1.41 tinted pale green; <0.71 tinted yellow; <0.50 tinted pink. Values where the difference between the observed and expected values is statistically significant at *p* < 0.05 are shown in bold type.

	MAM	HAE	GYN	PED	COL	CNS	HEN	LIV	LUN	STO	PRO
TR	**0.83**	**1.19**	**1.25**	**1.40**	**0.90**	**1.36**	**1.09**	**0.87**	**1.57**	**1.40**	**1.15**
IR	**1.24**	**0.86**	**0.78**	**0.74**	1.08	**0.78**	**0.90**	**0.57**	**0.52**	**1.13**	**0.87**
EG	**0.78**	0.93	**0.67**	0.99	**0.79**	**0.63**	**0.56**	**2.78**	**0.72**	**0.76**	**0.61**
MY	**1.53**	**0.73**	0.99	**0.42**	**1.69**	**0.57**	**1.28**	**0.72**	**0.58**	**0.41**	**0.71**
SA	1.07	**0.72**	**0.76**	**0.66**	**1.44**	0.96	**1.21**	**1.28**	0.82	**0.75**	**0.77**
PK	1.07	0.94	0.94	**0.64**	**0.57**	**0.71**	**1.46**	1.04	**0.57**	**0.52**	0.93
TN	0.89	1.00	0.86	0.98	**1.37**	1.05	0.93	0.84	1.23	0.96	**0.58**
MA	**0.82**	1.05	1.25	1.07	0.73	**1.50**	**1.45**	0.94	**1.67**	1.23	**0.38**
NG	**1.25**	**0.74**	**1.98**	**1.46**	**0.45**	**0.65**	**1.48**	**0.50**	**0.38**	**0.44**	**1.84**
LB	0.96	**2.10**	**0.39**	**1.66**	**1.51**	1.01	0.77	0.77	0.88	**1.59**	1.10
JO	**1.36**	1.11	0.65	**2.11**	1.33	1.06	0.85	**0.13**	**0.49**	**0.18**	0.97
ID	**1.37**	**0.61**	**1.61**	1.14	0.95	**0.45**	1.03	0.80	0.65	**0.32**	1.21
AE	**1.39**	0.74	0.94	0.85	0.96	0.74	0.71	1.08	0.79	1.16	**0.42**
KW	**1.43**	1.33	**0.49**	1.11	1.05	1.20	1.31	**0.41**	0.74	0.49	**0.43**
QA	1.31	1.19	1.42	**0.43**	1.30	0.78	**0.32**	0.68	0.65	0.52	1.16
**Total**	**6593**	**5416**	**3092**	**2985**	**2687**	**2525**	**2421**	**2229**	**2113**	**1990**	**1806**

**Table 3.** Relative output within cancer research of work on 10 research types (codes below Figure 5) by the leading 15 countries (see Table 1), 2008–17. Cells where this ratio >2.0 tinted green; >1.41 tinted pale green; <0.71 tinted yellow; <0.50 tinted pink. Values where the difference between the observed and expected values is statistically significant at *p* < 0.05 are shown in bold type.

	GENE	PROG	CHEM	SURG	PATH	EPID	DIAG	RADI	SCRE	CLINT
TR	**0.81**	**1.16**	**1.07**	**1.61**	0.99	**0.69**	**1.25**	**1.63**	**0.80**	**0.63**
IR	**1.26**	**0.78**	**1.26**	**0.50**	**0.82**	**1.11**	**0.81**	**0.55**	0.86	**1.20**
EG	1.05	**1.28**	**1.12**	0.97	**1.25**	**0.62**	**1.26**	0.87	**0.72**	**1.66**
MY	**1.13**	**0.65**	**0.73**	**0.46**	0.99	1.05	**0.59**	**0.41**	**1.91**	**0.66**
SA	1.03	**0.80**	1.02	**0.71**	1.06	0.88	**0.78**	**0.58**	1.20	**0.60**
PK	**0.88**	0.93	**0.79**	**0.70**	**1.19**	**1.32**	**1.23**	0.78	0.83	**0.29**
TN	**1.43**	0.93	**0.42**	**0.69**	**1.57**	**1.51**	**0.73**	0.95	0.70	0.60
MA	**0.70**	**0.36**	**0.42**	**1.71**	0.89	**0.67**	1.15	**1.45**	0.79	**0.31**
NG	**0.37**	**0.52**	**0.62**	1.16	0.77	**1.43**	1.15	1.13	**3.74**	0.41
LB	**0.68**	0.86	**1.30**	1.19	**0.63**	0.84	**0.52**	1.20	0.69	1.68
JO	0.89	0.91	0.86	0.98	1.00	1.40	1.04	0.92	1.27	**0.20**
ID	**1.43**	0.83	1.18	0.84	**0.59**	**1.42**	0.66	0.74	1.06	0.98
AE	1.02	0.98	1.09	0.68	1.19	1.10	0.86	0.64	1.15	0.42
KW	1.04	**0.38**	0.67	**0.46**	**2.05**	0.50	**2.12**	0.71	1.10	**0.10**
QA	0.76	1.02	0.64	0.84	0.53	1.45	0.70	0.55	2.11	0.80
**Total**	**8124**	**4804**	**4799**	**4652**	**3578**	**2633**	**2529**	**1923**	**1071**	**684**

**Table 4.** Numbers of funding acknowledgments to universities (UNIV) and government departments and agencies (GOV) in 10 leading OIC countries and to government (GOV) and private-non-profit funders (PNP, collecting charities and endowed foundations) in 10 leading non-OIC countries, on OIC cancer research papers, 2009–17.

OIC countries				Non-OIC countries			
**Country**	**ISO2**	**UNIV**	**GOV**	**Country**	**ISO2**	**GOV**	**PNP**
Iran	IR	3931	352	United States	US	3575	1130
Turkey	TR	1735	865	United Kingdom	UK	1024	1260
Malaysia	MY	1328	917	France	FR	625	352
Saudi Arabia	SA	1285	169	Germany	DE	534	346
Egypt	EG	241	230	Netherlands	NL	392	241
Pakistan	PK	105	278	Canada	CA	359	267
Tunisia	TN	21	330	Italy	IT	275	345
Lebanon	LB	116	65	Spain	ES	431	87
Indonesia	ID	51	77	Japan	JP	395	64
Bangladesh	BD	18	26	China	CN	388	0

**Table 5.** Citation performance (five-year, fractional country counts) for cancer research papers from the 15 leading OIC countries in terms of arithmetic mean values (ACI mean) and presence in the top 5% (29 cites or more). Cells where this ratio >1.41 tinted pale green; <0.71 tinted yellow; <0.50 tinted pink. Numbers of papers with 29+ cites that are significantly below the values expected shown in bold type. For country codes, see Table 1.

Country	Citable papers	Cites	ACI mean	Top 5%			W.S. 5%
				Expected	Observed	Sign.	
SA	917	8939	9.8	45.8	57.8	n.s.	125
QA	40	536	13.3	2.0	2.4	n.s.	117
AE	150	1399	9.3	7.5	8.6	n.s.	114
LB	259	2259	8.7	12.9	12.9	n.s.	99
ID	86	941	10.9	4.3	4.3	n.s.	98
EG	1615	15056	9.3	80.8	78.1	n.s.	96
JO	209	1546	7.4	10.5	9.3	n.s.	88
MY	1119	9438	8.4	56.0	49.6	n.s.	88
IR	3030	23722	7.8	151.5	**115.4**	0.25%	76
PK	785	4020	5.1	39.3	**15.1**	0.01%	38
NG	286	1524	5.3	14.3	**5.3**	1.50%	37
TR	7479	39326	5.3	373.9	**111.8**	0.00%	30
TN	630	3054	4.8	31.5	**7.7**	0.00%	24
KW	172	947	5.5	8.6	**2.1**	2.36%	24
MA	401	1348	3.4	20.1	**3.7**	0.01%	19

**Table 6.** The numbers of cancer researchers from five Levantine countries: United Arab Emirates (AE), Jordan (JO), Kuwait (KW), Oman (OM) and Qatar (QA) in 2015–17 from different ethnic or national backgrounds, based on the Origins database classification of names into ten geographical regions.

Countries	Code	AE	JO	KW	OM	QA	Total	%
Other Muslim countries	*MUS*	154	269	89	93	166	771	*39.4*
Levant including Turkey	*LEV*	74	207	41	40	102	464	*23.7*
Western Europe	*EUR*	60	29	23	13	80	205	*10.5*
Maghreb* + Egypt	*MED*	43	51	12	7	46	159	*8.1*
Bhutan, India, Nepal, Sri Lanka	*IND*	50	10	21	25	36	142	*7.3*
Africa (Sub-Saharan)	*AFR*	26	55	13	9	15	118	*6.0*
Eastern Europe incl. Russia	*EEU*	13	12	5	1	11	42	*2.1*
Not known	*UNK*	5	12	5	0	5	27	*1.4*
East Asia	*EAS*	9	1	0	0	5	15	*0.8*
Latin America	*LAT*	3	1	0	4	4	12	*0.6*
	*Total*	437	647	209	192	470	1955	

* The Maghreb consists of Algeria, Libya, Mauritania, Morocco and Tunisia.
